# Importance of Autophagy in Mediating Human Immunodeficiency Virus (HIV) and Morphine-Induced Metabolic Dysfunction and Inflammation in Human Astrocytes

**DOI:** 10.3390/v9080201

**Published:** 2017-07-28

**Authors:** Myosotys Rodriguez, Jessica Lapierre, Chet Raj Ojha, Hary Estrada-Bueno, Seth M. Dever, David A. Gewirtz, Fatah Kashanchi, Nazira El-Hage

**Affiliations:** 1 Department of Immunology, Herbert Wertheim College of Medicine, Florida International University, Miami, FL 33199, USA; myrodrig@fiu.edu (M.R.); jlapi008@fiu.edu (J.L.); cojha001@fiu.edu (C.R.O.); hestrada@fiu.edu (H.E.-B.); Seth.Dever@dgs.virginia.gov (S.M.D.); 2 Department of Pharmacology and Toxicology, School of Medicine, Virginia Commonwealth University, Richmond, VA 23298, USA; David.gewirtz@vcuhealth.org; 3 National Center for Biodefense and Infectious Diseases, George Mason University, Manassas, VA 20110, USA; fkashanc@gmu.edu

**Keywords:** autophagy, human immunodeficiency virus, neurotransmitters, inflammation, morphine, astrocytes

## Abstract

Under physiological conditions, the function of astrocytes in providing brain metabolic support is compromised under pathophysiological conditions caused by human immunodeficiency virus (HIV) and opioids. Herein, we examined the role of autophagy, a lysosomal degradation pathway important for cellular homeostasis and survival, as a potential regulatory mechanism during pathophysiological conditions in primary human astrocytes. Blocking autophagy with small interfering RNA (siRNA) targeting *BECN1*, but not the Autophagy-related 5 (*ATG5*) gene, caused a significant decrease in HIV and morphine-induced intracellular calcium release. On the contrary, inducing autophagy pharmacologically with rapamycin further enhanced calcium release and significantly reverted HIV and morphine-decreased glutamate uptake. Furthermore, siBeclin1 caused an increase in HIV-induced nitric oxide (NO) release, while viral-induced NO in astrocytes exposed to rapamycin was decreased. HIV replication was significantly attenuated in astrocytes transfected with siRNA while significantly induced in astrocytes exposed to rapamycin. Silencing with siBeclin1, but not siATG5, caused a significant decrease in HIV and morphine-induced interleukin (IL)-8 and tumor necrosis factor alpha (TNF-α) release, while secretion of IL-8 was significantly induced with rapamycin. Mechanistically, the effects of siBeclin1 in decreasing HIV-induced calcium release, viral replication, and viral-induced cytokine secretion were associated with a decrease in activation of the nuclear factor kappa B (NF-κB) pathway.

## 1. Introduction

Astrocytes are the most abundant cell type in the central nervous system (CNS) and contribute to a variety of tasks, ranging from buffering calcium release and glutamate uptake [1,2,3], to regulating brain immune response [4] and controlling the blood-brain barrier (BBB) and blood flow [5]. Furthermore, it is well accepted that astrocytes can maintain a low level of human immunodeficiency virus (HIV) infection [6,7,8,9] and develop into viral reservoirs that impede eradication, although the mechanisms that lead to HIV latency in this cell type are still not completely understood. During HIV neuroinflammation, infected astrocytes release inflammatory cytokines and chemokines, as well as neurotoxins that can subsequently cause neuronal damage and death [4,10]. Once the infected astrocytes no longer serve as metabolic support for neurons, HIV-induced neuronal loss is aggravated. Since astrocyte dysfunction is a severe neuropathological finding of HIV-associated encephalitis, it can be reasoned that astrocyte activation leads to impaired astrocyte-neuron networks, thus contributing to apoptosis of neurons [11]. It is now commonly understood that HIV-associated neurological disorders are caused in part by astrocytic dysfunction [12]. Further complication with opiate drug abuse (e.g., morphine), a frequent comorbidity of HIV infection [3,4,5,6,7,8,9,10,11,12,13,14], is known to amplify HIV-induced dysfunctions in astrocytes [13,14,15,16,17]. Morphine is the main bioactive product of heroin in the brain [18] and among the most frequently prescribed opioid for the treatment of severe pain in HIV and acquired immune deficiency syndrome (AIDS) patients, making the use of morphine for in vitro studies extremely relevant. Although evidence has proven the important role of astrocytes in HIV and morphine-induced neurological disorders, elucidating a potential molecular mechanism(s) by which HIV-infected astrocytes affect brain homeostasis and regulate neuro-inflammation are still of great interest.

The autophagy pathway engulfs and sequesters cytoplasmic proteins in a unique membranous compartment known as the autophagosome, for lysosomal degradation (reviewed in [19]). Autophagy is constitutively active in neurons and required for neuronal survival while a disruption in the pathway can affect the intercellular communication of axons, damage dendrites and synaptic structures, and subsequently contribute to neurodegenerative disorders such as Alzheimer’s disease and NeuroAIDS [20,21,22]. Specific autophagy-inducing agents are being considered for therapeutic treatment and prevention of a broad range of human diseases [20,21,23,24,25]. In the last decade, numerous lines of evidence have shown that autophagy is a critical target for HIV during the viral life cycle which has led to an increasing effort to understand the role of autophagy in those cells affected by HIV infection [26,27,28]. Beclin1 is part of a phosphatidylinositol 3-kinase complex that initiates the formation of the isolation membrane of the autophagosome in the autophagy pathway [19], and can be upregulated by pathogens to aid in replication [29]. Growing evidence suggests Beclin1 as a possible target for modulation of the autophagy pathway and subsequently decreasing inflammatory responses [30,31]. Our laboratory recently demonstrated the importance of Beclin1 in regulating viral replication and viral-induced inflammation in HIV-infected microglia exposed to morphine [32,33]. In addition to Beclin1, the autophagy-related protein 5 (ATG5) is involved in the initial stages of autophagosome formation. While less explored for its role in HIV induced neuropathologies, ATG5 has been linked to HIV infectivity mostly in macrophages [28,34].

The association of autophagy and astrocytes in other neuropathologies not involving HIV has been explored more extensively. For example, an Alzheimer’s disease model showed autophagy has a key role in amyloid internalization/uptake in glial cells [35]. In multiple sulfatase deficiency (MSD), a lysosomal storage disorder specifically induced in astrocytes, dysfunctional autophagy was reported in association with cortical damage suggesting a central role for astrocyte autophagic function in brain homeostasis [36]. Autophagy pathway has also been linked with synaptic transmission [37,38] and glutamate toxicity [39,40]. In fact, in vitro studies have shown that glutamate could also modulate the autophagy pathway [41,42]. Therefore, the connection between autophagy and glutamate uptake disruption due to HIV needs to be explored. More pertinent to our study, studies have shown a protective role of autophagy on astrocytes upon injury or HIV infection [43]. Also, a more recent report showed that the HIV protein gp120 in combination with methamphetamine induced autophagy in astrocytes [44]. It is therefore clear that the role of autophagy in HIV-induced astrocyte dysfunction, specifically in the context of opioid abuse, has not been extensively explored. In this study, we investigated the role of the autophagy pathway as a possible molecular mechanism responsible for regulating intracellular calcium release, glutamate uptake and release of reactive oxygen and nitrogen species in astrocytes during pathological conditions with HIV and morphine. We also examined the mechanistic role of autophagy in regulating HIV replication and HIV and morphine-induced inflammatory cytokines in astrocytes. Overall, the data provides new insight into mechanisms operating calcium release and glutamate uptake in astrocytes during pathological and normal physiological conditions and supports the role of autophagy as a possible mediator in regulating HIV replication and inflammatory cytokines in astrocytes infected with HIV and exposed to morphine.

## 2. Materials and Methods

### 2.1. HIV Infection, Treatments and Transfection of Human Astrocytes

Phytohemagglutinin (PHA)-activated peripheral blood mononuclear cells (PBMCs) were infected with HIV_SF162_ strain at concentrations of 100 ng/mL. The cell culture supernatants/conditioned media were harvested, filtered, and stored at −80 °C as performed in previous studies [45]. Viral stocks were quantified by assaying for HIV p24 (RETRO-TEK, Buffalo, NY, USA). Primary human astrocytes (ScienCell, Carlsbad, CA, USA) cultured in Astrocyte Medium (ScienCell) were grown to ~75–80% confluency and infected with the HIV_SF162_ strain (NIH AIDS Reagent, Germantown, MD, USA), as used in our previous publications [33,46,47]. Astrocytes were exposed to 1 ng/mL of HIV p24/10^6^ cells for up to 7–10 days to achieve restricted infection. Morphine (MOR) sulfate and rapamycin (RAP) were purchased from Sigma-Aldrich (St. Louis, MO, USA) and used at a concentration of 1 and 2.5 μM, respectively. Concentrations were chosen after performing dose-response viability assays. In some experiments, cells were transfected with control siRNA, Beclin1 siRNA or ATG5 siRNA as described previously [32], followed by exposure with HIV ± morphine at different time points as indicated in the text.

### 2.2. Assessment of Intracellular Calcium ([Ca^2+^]_i_)

Human astrocytes were loaded with 2.5 μM fura-2-AM (Invitrogen, Carlsbad, CA, USA) for 30 min at 37 °C, 5% CO_2_. Cells were washed three times with PBS and incubated in culture medium for an additional 30 min in order to achieve de-esterification of the AM group. After recording baseline measurements (20 s), astrocytes were exposed to HIV followed by respective treatments. Fura-2 ratio at 340/380 nm excitation measurements were taken every 10 s for a total of 810 s. BAPTA/AM at 20 µM was used to chelate calcium.

### 2.3. Glutamate Uptake Measurements

Glutamate uptake was measured in human astrocytes according to a previous study [16]. Cells were pre-incubated for 60 min at 37 °C with Hank’s balanced salt solution (HBSS) (Millipore, Billerica, MA, USA) with respective treatments. Glutamate at a concentration of 1 mM was added to each well. Sample supernatants were collected from individual wells at 0–240 min time points. Glutamate levels were quantified using a glutamate assay kit (BioVision, Mountain View, CA, USA) according to the manufacturer’s protocol. Briefly, 10 to 50 µL of supernatant was directly diluted in the assay buffer, and 100 µL of reaction mix was added to the samples and the glutamate standard. The reaction was incubated at 37 °C for 30 min. Optical density (O.D.) was measured at 450 nm and glutamate concentrations were calculated.

### 2.4. Reactive Oxygen Species Assay

Levels of reactive oxygen species (ROS) production were measured by using the indicator 5-(and-6)-chloromethyl-2′,7′-dichlorodihydrofluorescein diacetate, acetyl ester (CM-H2 DCFDA; Invitrogen), which is de-acetylated to dichlorofluorescein (DCF). Human astrocytes were loaded with 10 μM CM-H2 DCFDA in warm HBSS for 1 h according to the manufacturer’s protocol, then washed twice before treatments were applied. After 0, 1, 4 and 16 h of treatment, cells were incubated for different time points at 37 °C before fluorescence was measured at λ_ex_ = 485 nm and λ_em_ = 520 nm using a Synergy HTX plate reader (BioTek, Winooski, VT, USA).

### 2.5. Nitric Oxide Assay

Nitric oxide (NO) production by glial cells was measured using the Griess Reagent System (Promega, Madison, WI, USA), according with the manufacturer. Briefly, in a 96-well plate, 50 µL of culture supernatant was dispensed into the well in triplicate. After adding culture supernatants, 50 μL of the sulfanilamide solution was added to all the samples and incubated for 10 min at room temperature, protected from light. Following incubation, 50 μL of the *N*-1-napthylethylenediamine dihydrochloride (NED) solution was added to all the wells and incubated for an additional 10 min at room temperature, protected from light. Absorbance was measured within 30 min in a Synergy HTX plate reader (BioTek) with filters between 520 nm and 550 nm. Concentration of NO was calculated based on the standard curve using known concentrations of nitrite.

### 2.6. ELISA

Levels of interleukin (IL)-8, monocyte chemotactic protein-1 (MCP-1) and tumor necrosis factor alpha (TNF-α) were measured by ELISA 24 h post-treatment (R&D Systems, Minneapolis, MN, USA). Culture supernatants containing HIV particles were used to measure p24 protein levels by ELISA 24 h post-treatment according to the manufacturer’s protocol (RETRO-TEK). The O.D. was read at A450 on a Synergy HTX plate reader (BioTek). In addition, protein lysates from human astrocytes after 8 h treatment were used to measure NF-κB, c-Jun N-terminal kinase (JNK) and p38/MAPK protein expression by ELISA (Cell Signaling Technology, Danvers, MA, USA) according to the manufacturer’s instructions. The O.D. was read at A450 on a Synergy HTX plate reader (BioTek).

### 2.7. Immunoblotting

Whole cell lysates from human astrocytes were prepared in Radioimmunoprecipitation assay (RIPA) buffer supplemented with a mixture of protease and phosphatase inhibitors and separated by Sodium Dodecyl Sulfate Polyacrylamide Gel Electrophoresis (SDS-PAGE) for immunoblotting. Primary antibodies against NF-κB/p65 (1:200), p38 α/β (1:200) and JNK (detects isoforms p46 and p54) (1:200), were from Santa Cruz Biotechnology (Santa Cruz, CA, USA). Glyceraldehyde-3-phosphate dehydrogenase (GAPDH) (1:1000) was from Sigma-Aldrich. Primary antibodies were followed by incubation with a secondary antibody conjugated to horseradish peroxidase (Millipore, Billerica, MA, USA) used at a 1:1000 dilution. The immunoblots were exposed to SuperSignal West Femto Substrate (Thermo Scientific, Waltham, MA, USA) and visualized using a ChemiDoc imaging system (Bio-Rad, Hercules, CA, USA).

### 2.8. Statistical Analysis

Data were analyzed using analysis of variance (ANOVA) techniques followed by Bonferonni’s post hoc test for multiple comparisons (GraphPad Prism 6 software, Inc., La Jolla, CA, USA). An α level of *p* < 0.05 was considered significant.

## 3. Results

### 3.1. Role of Autophagy in Mediating HIV and Morphine-Induced Release of Intracellular Calcium ([Ca^2+^]_i_) and Glutamate Uptake in Astrocytes.

**Figure 1:**
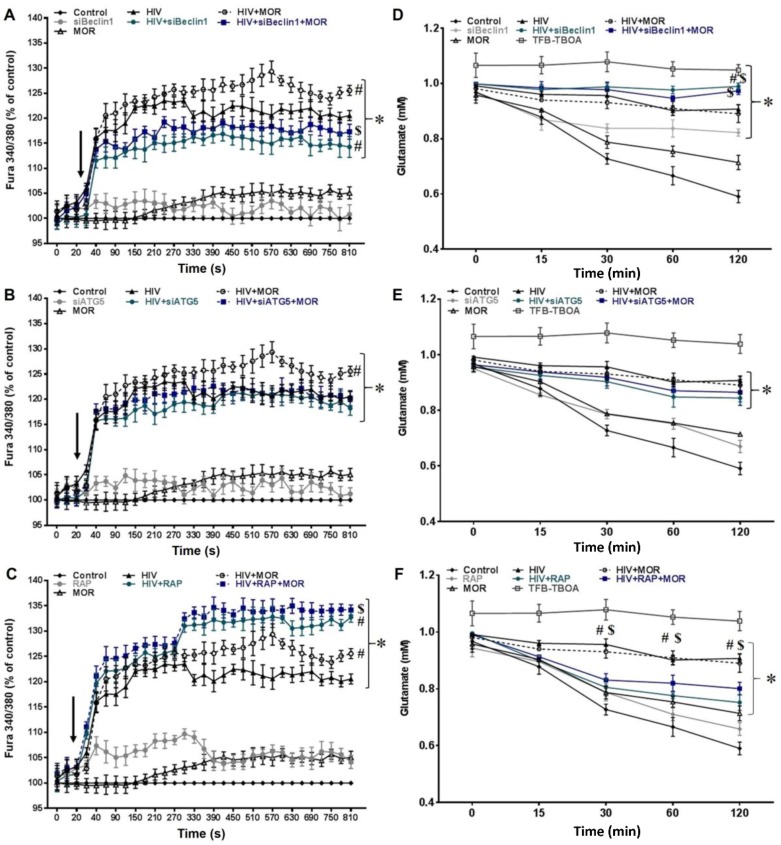
Intracellular calcium release and glutamate uptake are mediated by the autophagy pathway. (**A**–**C**) Effects of autophagy on [Ca^2+^]_i_ production in astrocyte cultures were assessed by fura-2AM at the indicated time points. Arrows represent the time of treatment with human immunodeficiency virus (HIV), morphine (MOR) alone (1 µM) or in combination, after 20 s of basal readings. Results represent the percentage of control values and are presented as the mean ± the standard error of the mean (SEM) of three independent experiments pre-treated with (**A**) siBeclin1, (**B**) siATG5 and (**C**) rapamycin in human astrocytes (*p* < 0.05 * vs. Control, ^#^ vs. HIV, ^$^ vs. HIV + MOR). (**D**–**F**) Human astrocytes infected with HIV alone or in combination with morphine were challenged with glutamate (1.0 mM) and levels of residual glutamate in the medium were measured at the indicated time points for experiments in which astrocytes were transfected with (**D**) siBeclin1, (**E**) siATG5 and pre-treated with (**F**) rapamycin. Gray line represents cells treated with the excitatory amino acid transporter inhibitor, (3S)-3-[[3-[[4-(Trifluoromethyl)benzoyl]amino]phenyl]methoxy]-l-aspartic acid (TFB-TBOA). Data are presented as the mean ± SEM of three independent experiments (*p* < 0.05 * vs. Control, ^#^ vs. HIV, ^$^ vs. HIV + MOR).

Increased intracellular calcium release by HIV and morphine in astrocytes can lead to neuronal injury [13,48,49]. However, the mechanism mediating this release is not fully discerned. The role of autophagy in mediating HIV and morphine-induced release of intracellular [Ca^2+^]_i_ was monitored using the fluorescent indicator Fura-2. Exposure to morphine (MOR) showed no significant changes in [Ca^2+^]_i_ when compared to uninfected control human astrocytes (Figure 1A–C, indicated by arrow). Exposure to HIV caused a significant release in [Ca^2+^]_i_ in human astrocytes when compared to control group and was significantly enhanced (at later time-points) when co-exposed to morphine (Figure 1A–C, indicated by arrow). It should be noted that exposure to HIV in a short time frame will not lead to active viral replication in astrocytes. While we cannot exclude Tat-mediated effects, exposure to HIV virions can also permit direct cell contact with the coat protein, gp120, which has been shown to be sufficient to cause increases in intracellular calcium [50,51,52,53]. Blocking autophagy by transfecting un-infected human astrocytes with siRNA against the *BECN1* gene (Figure 1A; gray line) showed no significant changes in the release of [Ca^2+^]_i_ when compared to control group; however, in astrocytes exposed to HIV alone (Figure 1A; green line) or in combination with morphine (Figure 1A; blue line), siBeclin1 caused a significant decrease in calcium release when compared to similar treatment in the absence of siBeclin1. To confirm whether this effect was specific to Beclin1, we silenced the autophagy protein ATG5 (Figure 1B; gray line) and showed no significant changes in the release of [Ca^2+^]_i_ when compared to control group, HIV alone (Figure 1B; green line) or in combination with morphine (Figure 1B; blue line). This data confirms the involvement of Beclin1 and not necessarily ATG5 in regulating calcium release. Inducing autophagy pharmacologically with rapamycin (RAP) (Figure 1C; gray line) showed no significant changes in the release of [Ca^2+^]_i_ when compared to control group; however, in astrocytes exposed to HIV alone (Figure 1C; green line) or in combination with morphine (Figure 1C; blue line), rapamycin caused a further increase in calcium release when compared to similar treatment in the absence of rapamycin. Rapamycin was used with caution since this chemical is known to inhibit the mammalian target of rapamycin (mTOR), interfering with the phosphoinositide 3-kinase (PI3K)-Akt-mTOR axis that is key to several cellular functions involving differentiation, viability and growth [54]. The optimum dose of 2.5 µM was decided based on the reduced dose response values and the effects on cell viability. Of note, the transfection efficiency of siBeclin1 and siATG5 and the efficacy of the pharmacological inhibitor, rapamycin, in astrocytes ± HIV was confirmed using an Autophagy Tandem Sensor red fluorescent protein (RFP)-green fluorescent protein (GFP)-LC3 based assay that monitors autophagosome formation and autophagic flux, while protein expression levels of LC3 and Beclin1 were confirmed by western blot analysis. Live dead assay showed that modulation of autophagy by gene silencing and pharmacological intervention did not affect human astrocyte viability (Supplementary data; Figure S1). To ensure that the increased Fura-2 ratios corresponded to intracellular calcium release, we pretreated astrocytes with the [Ca^2+^]_i_ chelator, BAPTA/AM. Pretreatment with BAPTA abrogated calcium levels in astrocytes, confirming that the increased levels in Fura-2 ratios were due to the release of [Ca^2+^]_i_.

Glutamate uptake dysfunction in astrocytes is considered an important hallmark in HIV-induced neurotoxicity [16]. It was reported that activation of autophagy with rapamycin and trehalose decreased the effects of glutamate-induced excitotoxicity in hippocampal neurons [55]. Here, we explored whether the autophagy pathway is involved in this cellular process affected by HIV. After challenging cells with an excess of glutamate, extracellular glutamate was rapidly depleted in the uninfected control astrocytes (Figure 1D–F), and exposure to morphine showed no significant changes in glutamate uptake when compared to control group (Figure 1D–F). On the contrary, glutamate buffering was significantly attenuated in HIV-infected human astrocytes when compared to control group, while co-exposure to morphine showed no significant interactive effect (Figure 1D–F). Blocking autophagy with siRNA against the *BECN1* gene (Figure 1D; gray line) showed a decrease in glutamate uptake when compared to control group; however, in astrocytes exposed to HIV alone (Figure 1D; green line) or in combination with morphine (Figure 1D; blue line), siBeclin1 caused no significant changes (except at 120 min). Blocking autophagy with siATG5 (Figure 1E; gray line) showed no significant differences in glutamate uptake when compared to control group or in astrocytes exposed to HIV alone (Figure 1E; green line) or in combination with morphine (Figure 1E; blue line). Inducing autophagy with rapamycin (Figure 1F; gray line) showed no significant changes in glutamate uptake when compared to control group; however, in astrocytes exposed to HIV alone (Figure 1F; green line) or in combination with morphine (Figure 1F; blue line), rapamycin caused an increase in glutamate uptake when compared to similar treatment in the absence of rapamycin. In some experiments, cells were treated with the excitatory amino acid transporter EAAT1 (GLAST) and EAAT2 (GLT-1) inhibitor, TFB-TBOA, which showed strong inhibition in glutamate uptake, but not a significant increase in glutamate levels in the culture medium (Figure 1D–F). This suggests that decreased levels of glutamate in the medium were due to changes in buffering of glutamate molecules that were mediated through changes in the glutamate transporters EAAT1 (GLAST) and EAAT2 (GLT-1) function expressed on astrocytes. Overall, the data shows the specific role of Beclin1 in regulating calcium release and of rapamycin in regulating glutamate uptake in astrocytes under pathological conditions.

### 3.2. Role of Autophagy in Mediating HIV and Morphine-Induced Release of Oxyradicals in Astrocytes

**Figure 2:**
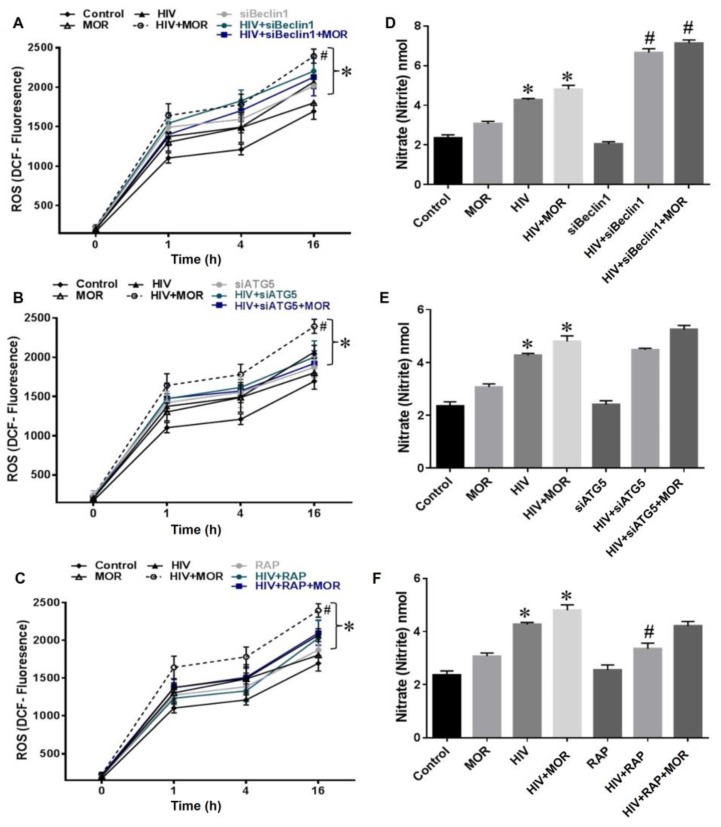
Limited effect of autophagy on reactive oxygen and nitrogen species. (**A**–**C**) The effect of autophagy on reactive oxygen species (ROS) production in human astrocytes infected with HIV alone or in combination with morphine (1 µM) was assessed by dichlorofluorescein (DCF) fluorescence at the indicated time points for experiments transfected (**A**) siBeclin1, (**B**) siATG5 and pre-treated with (**C**) rapamycin. (**D**–**F**) Reactive nitrogen species (RNS) release was measured by Griess Reagent System for experiments transfected (**D**) siBeclin1, (**E**) siATG5 and pre-treated with (**F**) rapamycin. Data are presented as the mean ± SEM of three independent experiments (*p* < 0.05 * vs. Control, ^#^ vs. HIV).

We next examined the role of autophagy in the production of Reactive Oxygen Species (ROS) in human astrocytes. ROS production has been tightly correlated with astrocyte dysfunction [12]. ROS release was assessed by DCF reactivity and showed no significant increase in astrocytes exposed to morphine (Figure 2A–C), when compared to control group. Infection with HIV caused an increase in ROS production while co-exposure to morphine significantly enhanced HIV-induced ROS production (Figure 2A–C). Blocking autophagy with siBeclin1 (Figure 2A; gray line) showed increased ROS production when compared to control group, while in astrocytes exposed to HIV alone (Figure 2A; green line) or in combination with morphine (Figure 2A; blue line), siBeclin1 caused no significant effect. Likewise, astrocytes transfected with siATG5 (Figure 2B; gray line) or treated with rapamycin (Figure 2C; gray line) caused no significant changes in ROS production when compared to control group. Furthermore, siATG5 and rapamycin caused no significant effect in astrocytes exposed to HIV alone (Figure 2B,C; green line) or in combination with morphine (Figure 2B,C; blue line).

Reactive nitrogen species (RNS) release assessed by Griess Reagent System showed no significant increase in RNS level in astrocytes exposed to morphine (Figure 2D–F), while RNS level in astrocytes infected with HIV alone or in combination with morphine was significantly increased when compared to control or morphine group (Figure 2D–F). Blocking autophagy with siBeclin1 (Figure 2D) showed no significant effect in RNS release when compared to control group, while in astrocytes exposed to HIV alone (Figure 2D) or in combination with morphine (Figure 2D), siBeclin1 caused a significant increase in RNS when compared to HIV-infected group without siRNA. Blocking autophagy in astrocytes with siATG5 (Figure 2E) caused no significant changes in RNS production when compared to control group. Likewise, siATG5 caused no significant effect in astrocytes exposed to HIV alone (Figure 2E) or in combination with morphine (Figure 2E). Inducing autophagy with rapamycin (Figure 2F) showed no significant changes in the release of RNS when compared to control group; however, in astrocytes exposed to HIV alone (Figure 2F), rapamycin caused a minimal, albeit significant decrease in RNS release when compared to HIV-infected group in the absence of rapamycin. Overall, the data shows that autophagy might not have a significant role in regulating RNS and ROS production in astrocytes under pathological conditions.

### 3.3. Role of Autophagy in Mediating HIV Replication and HIV and Morphine-Induced Inflammation in Astrocytes

**Figure 3:**
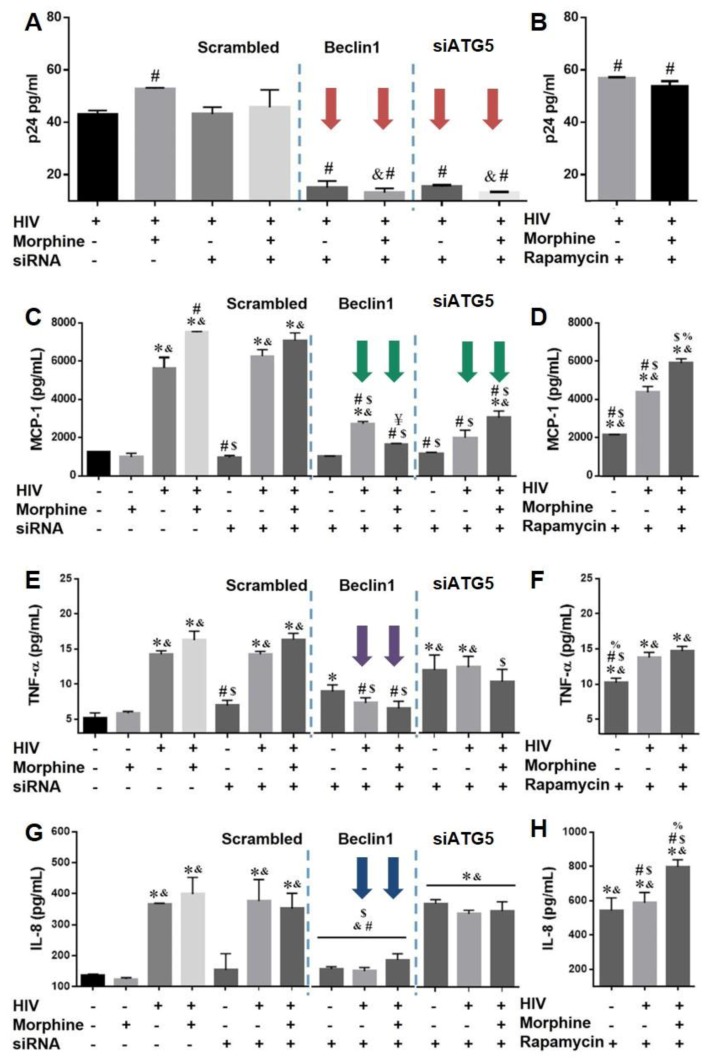
HIV replication and induced inflammation are mediated by the autophagy pathway. (**A**,**B**) HIV replication in human astrocytes infected with HIV alone or in combination with morphine (1 µM) was measured using HIV p24 Gag protein ELISA. Values were determined from standard curves and are presented as the mean ± SEM of three independent experiments (*p* < 0.05 ^#^ vs. HIV, ^&^ vs. HIV + MOR). (**D**–**H**) Corresponding cell culture supernatants were also used to detect the levels of pro-inflammatory cytokines (**C**,**D**) monocyte chemotactic protein-1 (MCP-1), (**E**,**F**) tumor necrosis factor alpha (TNF-α) (**G**,**H**) and interleukin (IL)-8 by ELISA. Values were determined from standard curves and are presented as the mean ± the SEM of three independent experiments (*p* < 0.05 * vs. Control, ^&^ vs. MOR, ^#^ vs. HIV, ^$^ vs. HIV + MOR, ^%^ vs. HIV + RAP, ^¥^ vs. HIV + siBeclin1).

We previously showed that silencing Beclin1 leads to the inhibition of HIV replication and HIV-induced monocyte chemotactic protein 1(MCP-1), regulated upon activation normal T cell expressed and presumably secreted (RANTES) and tumor necrosis factor alpha (TNF-α) responses in primary human microglia [32]. It has been well established that these inflammatory molecules are strongly linked with neuroinflammation by HIV [15,56,57]. As astrocytes undergo HIV infection, albeit to a lesser extent, we assessed whether autophagy modulation on HIV replication depends on cell-type specificity. Virus production in astrocyte supernatant measured by p24 Gag protein showed a slight, yet significant, increase in viral replication in HIV-infected astrocytes after 24 h co-exposure with morphine (Figure 3A). Blocking the autophagy pathway with siBeclin1 or siATG5 (Figure 3A; red arrows) in astrocytes infected with HIV alone followed by exposure with morphine caused an approximately 60% decrease in viral replication. To exclude the possibility that these effects were not specific to the modulation of autophagy by siRNA, we performed a control experiment using a scrambled non-silencing siRNA. As expected, transfection with scrambled control siRNA did not cause a significant change in virus production (Figure 3A), suggesting that changes in viral replication are mediated by targeting genes in the autophagy pathway and not by the transfection process itself. On the other hand, increasing the autophagy pathway with rapamycin in astrocytes infected with HIV followed by exposure with morphine showed a significant increase of approximately 33% in virus production (Figure 3B). Morphine exposure alone had no significant effect on cytokine secretion when compared to control uninfected astrocytes (Figure 3 and Supplementary Data; Figure S2). HIV infection alone caused a significant increase in the release of MCP-1, TNF-α and interleukin (IL)-8 when compared to control group which was further enhanced in combination with morphine (Figure 3C,E,G). Blocking autophagy with siBeclin1 or siATG5 (Figure 3C, green arrows) and inducing the pathway with rapamycin (Figure 3D) in astrocytes infected with HIV alone or in combination with morphine caused a significant decrease in MCP-1 secretion, albeit inhibition was more significant and robust with siRNAs. Interestingly, siBeclin1 transfected astrocytes infected with HIV alone or in combination with morphine caused a significant decrease in TNF-α (Figure 3E; purple arrows) release by 50%, while no significant attenuation in TNF-α was detected with siATG5 (Figure 3E) and rapamycin (Figure 3F). Furthermore, siBeclin1 transfected into astrocytes infected with HIV alone or in combination with morphine caused a significant decrease in IL-8 (Figure 3G; blue arrows) release by 63%, yet no significant attenuation in IL-8 was detected with siATG5 (Figure 3G) while exposure to rapamycin markedly increase in IL-8 release (Figure 3H). In addition, siATG5 and rapamycin treated astrocytes caused a significant increase in TNF-α (Figure 3E,F) and IL-8 (Figure 3G,H) release when compared to control treated astrocytes. In summary, the data shows that autophagy plays a role in viral replication in astrocytes, and supports the involvement of the protein Beclin1 in regulating TNF-α and IL-8 release in astrocytes under pathological condition with HIV and morphine exposure.

### 3.4. Potential Mechanism(s) Linking the Autophagy Pathway with HIV Replication and Viral-Induced Inflammation in Astrocytes

**Figure 4:**
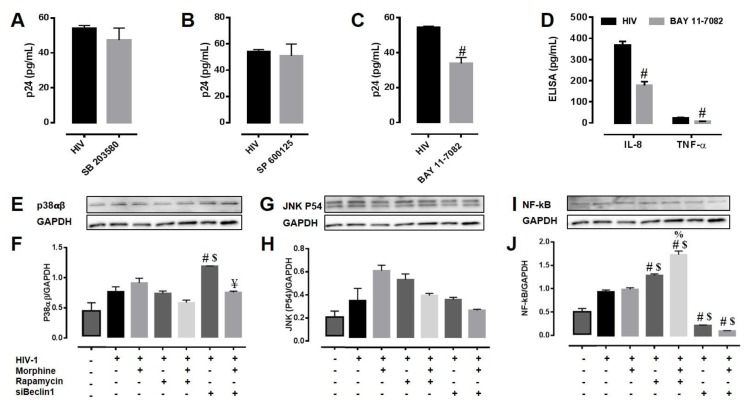
Autophagy-induced HIV replication and inflammation involves Mitogen activated protein kinases (MAPK) and nuclear factor kappa B (NF-κB) pathway. (**A**–**C**) HIV virus production in culture media from HIV infected human astrocytes pre-treated with the inhibitors (**A**) SB 203580 and (**B**) SP 600125 and (**C**) BAY 11-7082 for 24 h were measured using HIV p24 Gag protein ELISA. Values were determined from standard curves and are presented as the mean ± the SEM of three independent experiments (*p* < 0.05 ^#^ vs. HIV). (**D**) Corresponding cell culture supernatants were also used to detect the levels of pro-inflammatory cytokines IL-8 and TNF-α by ELISA. Values were determined from standard curves and are presented as the mean ± the SEM of three independent experiments (*p* < 0.05 ^#^ vs. HIV). (**E**–**J**) Cell lysates from HIV infected astrocytes with the indicated treatments were subjected to immunoblotting with antibodies to (**E**,**F**) p38, (**G**,**H**) c-Jun N-terminal kinase (JNK) and (**I**,**J**) nuclear factor kappa B (NF-κB). Densitometry was performed for quantification, and the ratios of each protein to Glyceraldehyde-3-phosphate dehydrogenase (GAPDH) are presented graphically. Error bars show the SEM of three independent experiments (*p* < 0.05 ^#^ vs. HIV, ^$^ vs. HIV + MOR, ^%^ vs. HIV + RAP, ^¥^ vs. HIV + siBeclin1).

The Mitogen activated protein kinases (MAPK’s) play an important role in regulating HIV replication and inflammatory responses [58,59,60]. To determine the role of p38 MAPK and JNK in the modulation of HIV replication and viral-induced inflammation in astrocytes by autophagy, we pre-treated the cells with chemical inhibitors targeting the p38 MAP kinase (SB 203580) and the JNK (SP 600125) pathways. Optimum dose of each inhibitor was decided based on its IC50 values and the effects on cell viability. Twenty-four hour exposure to SB 203580 and SP 600125 in astrocytes infected with HIV did not affect viral replication as detected by HIV-p24 Gag protein ELISA (Figure 4A,B). NF-κB has been extensively linked to the regulation of expression of inflammatory genes and HIV replication. To understand the involvement of NF-κB in the modulation of HIV replication and viral-induced inflammation in astrocytes by autophagy, we inhibited the cells with the inhibitor for NF-κB (Bay-11-7082) pathway. Treatment with Bay-11-7082 in astrocytes infected with HIV caused a significant decrease in viral replication after 24 h (Figure 4C), and significantly reduced levels of secreted TNF-α (34.6 ± 1.9%) and IL-8 (51.7 ± 2.4%) proteins (Figure 4D). We then explored the role of autophagy in regulating protein expression levels of MAPKs (p38 and JNK) and NF-κB. Twenty-four hour post-exposure to rapamycin in astrocytes infected with HIV alone or in combination with morphine showed no significant changes in p38 protein levels when compared to similarly treated lysates without rapamycin, while transfection with siBeclin1 caused an increase in protein expression that was reverted in combination with morphine (Figure 4E,F). Expression levels of JNK protein remained at basal levels and modulating the autophagy pathway with siBeclin1 and rapamycin did not affect protein expression in HIV-infected astrocytes (Figure 4G,H). On the contrary, exposure to rapamycin in astrocytes infected with HIV alone or in combination with morphine showed a marked increase in NF-κB (p65) expression levels when compared to similar treatments without rapamycin, while blocking the autophagy pathway with siBeclin1 caused a significant decrease in NF-κB (p65) protein levels in lysates that were similarly treated without siRNA (Figure 4I,J). To explore further whether the effect of these molecules would vary in a time-dependent manner, we repeated the study using an earlier time point (8 h) and analyzed protein expression by an ELISA assay which measures all three proteins (p38, JNK and NF-κB) in one assay and we were only able to detect a similar trend of NF-κB protein expression when compared with the 24 h time point (data not shown). In summary, rapamycin caused an increase in NF-κB expression which correlates with increased viral replication and viral-induced IL-8 in astrocytes (Figure 3), whereas siBeclin1 caused a decrease in NF-κB expression level which correlates with the decreased viral replication and viral-induced IL-8 and TNF-α in astrocytes (Figure 3). Overall, the data shows a mechanistic role of JNK and p38 MAPK proteins in regulating HIV-induced inflammatory molecules, but not necessary in regulating viral replication in human astrocytes. The data also provides a possible linkage between autophagy and NF-κB.

## 4. Discussion

**Figure 5:**
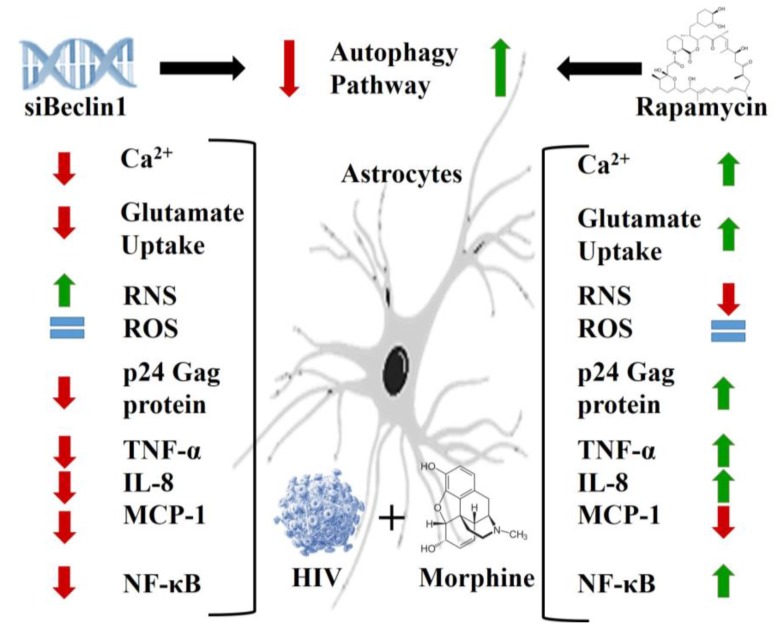
Proposed model for the role of autophagy in HIV-induced pathology and HIV-induced metabolic dysfunction in astrocytes in the context of opioid abuse. Green arrows indicate an induction, red arrows indicate a reduction, and blue equal symbols indicate no significant role of the respective molecules. Blocking the autophagy pathway with siBeclin1 significantly decreased HIV and morphine-induced intracellular calcium release and glutamate uptake with minimal changes in RNS and ROS production. Silencing with siBeclin1 significantly decreased HIV production and HIV and morphine-induced cytokines and chemokine secretion. On the contrary, increasing autophagy with rapamycin significantly increased HIV and morphine-induced intracellular calcium release, restored glutamate uptake and showed minimal decrease in RNS and ROS production. Rapamycin increased HIV replication, and HIV and morphine-induced TNF-α and IL-8 release. These effects correlate with the modulation of the NF-κB pathway by the autophagy pathway.

In this study, we investigated the role of autophagy as a possible molecular mechanism responsible for regulating intracellular calcium release, glutamate uptake and release of reactive oxygen and nitrogen species in astrocytes during HIV infection and morphine exposure. We also examined the mechanistic role of autophagy in regulating HIV replication and HIV and morphine-induced inflammatory cytokines in astrocytes. Using both gene silencing and small molecules inhibitors, we showed the specific role of Beclin1 in regulating calcium release and of rapamycin in regulating glutamate uptake in astrocytes under pathological conditions (Figure 1). Astrocytes have long been considered the provider of trophic support for neurons as they exhibit a form of excitability and communication on the basis of intracellular Ca^2+^ variations [61,62] that can be initiated by neuronal activity [63,64]. Ca^2+^ regulates neuronal plasticity underlying learning and memory and neuronal survival. Dysregulation of Ca^2+^ is decisive for brain cell death and degeneration [65,66], and Ca^2+^ elevation in astrocytes induces the release of glutamate [67,68,69]. Therefore, understanding the underlying molecular processes governing the initiation and propagation of astrocytic [Ca^2+^]_i_ waves is of critical importance for the development of novel therapeutic strategies to prevent neurodegeneration and confer neuroprotection. Glutamate release evokes a slow inward current in neurons and modulates action potential-evoked synaptic transmission between cultured hippocampal cells [68], suggesting that astrocytes may function within a network with bidirectional crosstalk with neurons. In the brain, glutamate serves as the main excitatory neurotransmitter; therefore, elevated levels of glutamate can contribute to neuronal injury and must be tightly regulated and kept at low levels [2,70,71]. Although the evidence for HIV-induced glutamate uptake disruption in terms of autophagy is very limited, evidence regarding other neurological disorders have shown a link between autophagy and glutamate uptake. For example, a study on Huntington's disease (HD) showed that increasing autophagy with rapamycin could restore glutamate uptake function in primary astrocytes from rat pups expressing the mutant Huntingtin (Htt-552) protein, which is commonly found in the brains of HD patients [72]. When we explored the role of autophagy on the disruption of glutamate uptake by HIV-exposed astrocytes, we did indeed detect a disruption in glutamate buffering by the HIV infection, with minimal added effect due to morphine (Figure 1D–F). Interestingly, our results showed similar trends as the study from Chen’s group on HD, in which pretreatment with rapamycin before a challenge of excess glutamate significantly restored glutamate uptake; whereas silencing with siBeclin1, but not siATG5, further decreased glutamate uptake in HIV infected astrocytes at a later time point. While assessing the role of autophagy in oxidative stress, we measured RNS production and saw that increasing autophagy by rapamycin treatment reversed HIV-induced NO release by astrocytes (Figure 2F). Concurring with other studies, a decreased rate in autophagy aggravated the toxic insult of the virus [44]. We were able to detect a similar trend, albeit not significant in the modulation of HIV-induced ROS release by the autophagy pathway (Figure 2A–C). We have previously shown that activation of the host autophagic pathway by HIV infection represents an essential mechanism in controlling viral replication and viral-induced inflammatory responses in microglial cells [32,33], although the interaction with morphine in terms of viral replication was mediated via an autophagy-independent manner in microglial cells [32]. Here, we showed that the combined effects of morphine on HIV replication and viral-induced inflammatory responses in astrocytes were mediated through a Beclin1-dependent mechanism. The discrepancy in the result could account for the inherent differences in the μ-opioid receptors (MOR) expressed and in the different level of viral infection between the two cell types. Astrocytes are known to undergo limited HIV infection compared to microglia and macrophages [7]. Here, we infected human astrocytes for 7–10 days to achieve a restricted infection and autophagy was induced with rapamycin and silenced with siBeclin1 and siATG5. Rapamycin has been previously proposed as a potential modulator of HIV entry because of its ability to downregulate C-C motif chemokine receptor 5 (CCR5) co-receptor expression in T-cells and macrophages [73]. On the other hand, a recent study showed no significant effect of rapamycin on HIV infectivity of human fetal astrocytes [43]. Contrasting to the results from Mehla and Chauhan studies [43], we were able to detect an increase in p24 by rapamycin treatment, though this may be attributed to variations in HIV strains, treatment concentration, and/or duration between both studies. Of note, a downregulation in CCR5 will principally affect HIV entry into the cell, rather than viral replication. Concurring with viral production, inducing autophagy with rapamycin significantly increased IL-8, while inhibiting the autophagy pathway with siBeclin1 and not with siATG5 significantly decreased the release of IL-8. This finding agrees with our past report where silencing of Beclin1 following HIV infection significantly decreased HIV production and HIV-induced IL-8 secretions in human microglia [33]. The release of IL-8 has been associated with blood brain barrier breakage and subsequently polymorphonuclear leukocyte recruitment [74], monocyte migration to sites of inflammation [75] and adhesion [76]. It has been widely reported that HIV and HIV proteins increases levels of IL-8 [77,78,79,80,81]. Moreover, IL-8 levels in the cerebrospinal fluid of HIV-associated dementia (HAD) patients are higher than HIV seropositive patients without neurological impairment [81]. Although the decrease in MCP-1 by rapamycin was minimal, it was significant and unexpected. Ongoing studies in our laboratory are further investigating whether this singularity was due to rapamycin modulating a kinase pathway different from the ones that were measured in this study. Mechanistically, the effects of siBeclin1 and rapamycin were directly associated with a respective decrease and increase in NF-κB activation (Figure 4). A decrease in NF-κB expression levels by siBeclin1 agrees with the detected decrease in virus production and in the inflammatory cytokines IL-8 and TNF-α (Figure 3). Importantly, not only can NF-κB play a role in orchestrating inflammatory responses in HIV infection [58,59,82], but increases in intracellular calcium release may cause NF-κB activation and subsequently cytokine, chemokine, and ROS production, which can exacerbate neuropathology [83,84]. In fact, several reports have shown a strong interlink between the autophagy and NF-κB pathways [85,86]. It is worth mentioning that while confirming modulation of autophagy-related genes and proteins by our treatments, we also detected a significant increase in several apoptotic genes when silencing Beclin1 which were further enhanced in the presence of morphine. Several reports have established that a modulation in autophagy could lead to an increase in programmed cell death [29,87,88]. In relation to HIV, a recent report showed that autophagy inhibition exacerbated gp120-methamphetamine-induced cell death [44]. Exposure to rapamycin in HIV-infected astrocytes caused a marked increase in the number of cells expressing both yellow and red puncta, compared to HIV-infected astrocytes without rapamycin, suggesting an increase in autophagosomes and in autophagic flux. On the contrary, siBeclin1 and siATG5 transfected into HIV-infected astrocytes caused a significant decrease in both yellow and red puncta, compared to HIV-infected astrocytes without siRNA, suggesting a decrease in autophagosomes and in autophagic flux. Exposure to rapamycin in astrocytes with or without HIV infection showed increased levels of LC3-I, LC3-II and Beclin1 protein expression, while silencing with siBeclin1 and siATG5 showed decreased levels of LC3-I, LC3-II and Beclin1 protein expression, compared to control group. Co-exposure to morphine showed a slight, albeit not significant, interactive effect in autophagy protein expression (Supplementary Data; Figures S1 and S2). While we did detect an increase in some apoptotic genes (Supplementary Data; Table S1), we were not able to detect any significant changes in cell viability (Supplementary Data; Figure S1). In summary, we report that autophagy could play a role in astrocyte functionality, specifically that HIV and morphine-induced pathology is mediated through a Beclin1-dependent mechanism, and this involvement is linked tightly with the NF-κB pathway (Figure 5). Although the role of autophagy in mediating HIV and morphine-induced metabolic dysfunction in human astrocytes seems to be modest, our study provides new insights on how modulating the autophagy pathway can influence HIV replication and HIV-induced pathology in human astrocytes in the context of opioid abuse.

## Supplementary Materials

The following are available online at www.mdpi.com/1999-4915/9/8/201/s1, Figure S1: Activation of the Autophagy pathway following gene silencing and pharmacological inducer, Figure S2: Effect of morphine on Beclin1 expression and inflammation in human astrocytes, Table S1: Treatment with rapamycin and transfection with siBeclin1 modulate cell survival-related gene.
