# Supplementary material for: Importance of Autophagy in Mediating Human Immunodeficiency Virus (HIV) and Morphine-Induced Metabolic Dysfunction and Inflammation in Human Astrocytes
Source: Viruses. 2017 Jul 28;9(8):201. doi: 10.3390/v9080201 (PMC5580458; doi:10.3390/v9080201)
Supplement: Supplementary file 1 [file viruses-09-00201-s001.pdf]

Importance of autophagy in mediating HIV and morphine-induced metabolic dysfunction and inflammation in human astrocytes

Myosotys Rodriguez[1], Jessica Lapierre[1], Chet Raj Ojha[1], Hary Estrada-Bueno[1], Seth M. Dever[1], David A. Gewirtz [2], Fatah Kashanchi [3], Nazira El-Hage[1]*

1: Department of Immunology, Florida International University, Herbert Wertheim College of Medicine, Miami, FL 33199, USA

2: Department of Pharmacology and Toxicology, Virginia Commonwealth University, School of Medicine, Richmond, VA 23298, USA

3: National Center for Biodefense and Infectious Diseases, George Mason University, Manassas, VA, 20110, USA

SUPPLEMENTAL MATERIAL

MATERIALS AND METHODS

HIV infection and treatments of human astrocytes

Primary human astrocytes (ScienCell, Carlsbad, CA, USA) cultured in Astrocyte Medium (ScienCell) were grown to ~75-80 % confluency followed by infection with HIV_{SF162} (NIH AIDS Reagent, Germantown, MD, USA) using 1 ng/mL of HIV p24/10^6 cells for up to 7-10 days, to achieve a latent or non-productive infection. After infection, cells were treated with 500 nM or 1 µM morphine sulfate (Sigma-Aldrich, St. Louis, MO, USA). After 8, 24 and 48 hours, culture supernatants were collected for further analysis.

Autophagy Flux Sensor

Autophagic flux was monitored using the Premo™ Autophagy Tandem Sensor RFP-GFP-LC3B Kit (Thermo Fisher Scientific, Grand Island, NY, USA) following the manufacturer's instructions. Cells were treated with 10 µL of BacMam reagents containing the RFP-GFP-LC3B construct for 12 hours. After incubation, cells were washed with PBS and mounted with ProLong® Gold Antifade Mountant (Thermo Fisher Scientific). DAPI staining was used to label cell nuclei. Images were analyzed for green fluorescence (LC3B positive autophagosomes) and red fluorescence

(autophagolysosome formation) using a Zeiss inverted fluorescence microscope with a 560 Axiovision camera.

Immunoblotting

Whole cell lysates from human astrocytes were prepared in RIPA buffer supplemented with a mixture of protease and phosphatase inhibitors and separated by SDS-PAGE for immunoblotting. Primary antibodies against Beclin1 (1:500) and LC3B (1:1000), were from Novus Biologicals (Littleton, CO, USA). Primary antibody against β-actin (1:200), was from Santa Cruz Biotechnology. Primary antibodies were followed by incubation with a secondary antibody conjugated to horseradish peroxidase (Millipore, Billerica, MA, USA) used at a 1:1000 dilution. The immunoblots were exposed to SuperSignal West Femto Substrate (Thermo Scientific, Waltham, MA, USA) and visualized using a ChemiDoc imaging system (Bio-Rad, Hercules, CA, USA).

Cell viability assay

Viability of human astrocytes was assessed using a live/dead cell fluorescence assay which combines fluorescent reagents to yield two-color discrimination of the population of live cells indicated by green fluorescence from the dead-cell population indicated by red fluorescence (ScienCell). Cells were imaged using a Zeiss inverted fluorescence microscope and viable cells were manually quantified and reported as percent of viability.

Autophagy RT^2 Profiler PCR Array

Autophagy-related gene expression was assessed after 24 hours treatment by mRNA isolation using the miRNeasy Mini Kit (Qiagen, Valencia, CA, USA). Purity of the RNA was measured by a microspot RNA reader (Synergy HT Multi-Mode Microplate Reader from BioTek) and RNA preparations with an O.D. 260/280 nm absorbance ratio of at least 2.0 were used for cDNA synthesis. One microgram of RNA was used for the first strand cDNA synthesis using Qiagen's RT2 First Strand Kit (catalog # 330401) as per the supplier's protocol. A genomic DNA elimination step was performed before reverse transcription. The Human Autophagy RT^2 Profiler PCR Array (Qiagen) profiles the expression of 84 key genes involved in autophagy. Relative abundance of each mRNA species was assessed using RT^2 SYBR Green/ROX PCR master mix (Qiagen; catalog

330520) containing 0.5 μg of RNA aliquoted in equal volumes (25 μL) to each well of the real-time PCR array using a Stratagene MX3000P qPCR system (Santa Clara, CA, USA). The threshold cycle (Ct) of each gene was determined by using Stratagene MaxPro software. The threshold and baseline were set manually according to the manufacturer's instructions. Ct data were uploaded into the data analysis template on the manufacturer's website (http://pcrdataanalysis.sabiosciences.com/pcr/arrayanalysis.php). The relative expression of each gene was calculated using the ΔΔCT method with five housekeeping genes and compared with the expression in control cells.

RESULTS

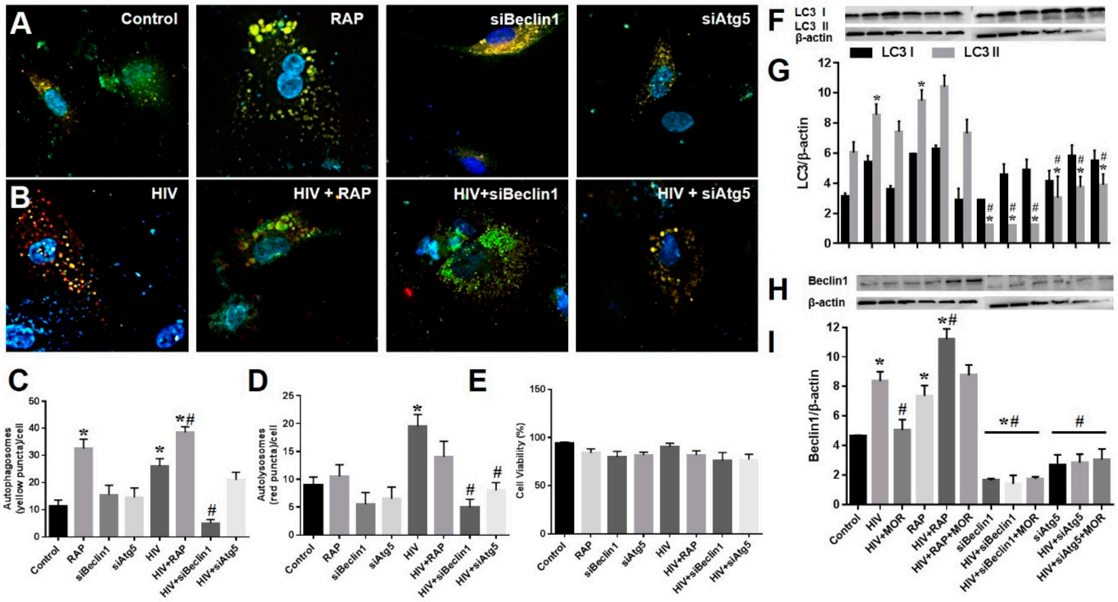

Figure 1. Activation of the Autophagy pathway following gene silencing and pharmacological inducer. (A, B) Representative images of astrocytes transfected with the Autophagy Tandem Sensor RFP-GFP-LC3 construct to monitor autophagosome and autophagic flux 24 hours post-treatments. Green fluorescence (GFP) is indicative of neutral pH autophagosomes, red fluorescence (RFP) is indicative of acidic pH autolysosomes and yellow fluorescence is indicative autophagosomes by colocalization (GFP+RFP). DAPI (blue) staining indicates cell nuclei. Images in panel A and B were acquired at 40X magnification. **(C)** Quantification of autophagosomes (yellow; GFP^{+} RFP^{+} LC3) and **(D)** autolysosomes (red; GFP^{-} RFP^{+} LC3) per cell from panels A and B. The data are presented as the average number of puncta per cell (± the SEM from three independent experiments) ($p<0.05^{*}$vs. Control, $^{\#}$vs. HIV). **(E)** Cell viability assay performed 24 hours after the indicated treatments showed no significant cellular toxicity. Data are presented as percentages of viable cells ± the standard error of the mean (S.E.M.) from three independent experiments. **(F-H)** Cell lysates were subjected to immunoblotting with antibodies to the indicated proteins. β-actin was used as a loading control. Densitometry was performed for quantification, and the ratios of each protein to GAPDH are presented graphically. Error bars show the S.E.M. of three independent experiments ($p<0.05$ *vs. Control, $^{\#}$ vs. HIV, $^{\$}$vs. HIV+MOR).

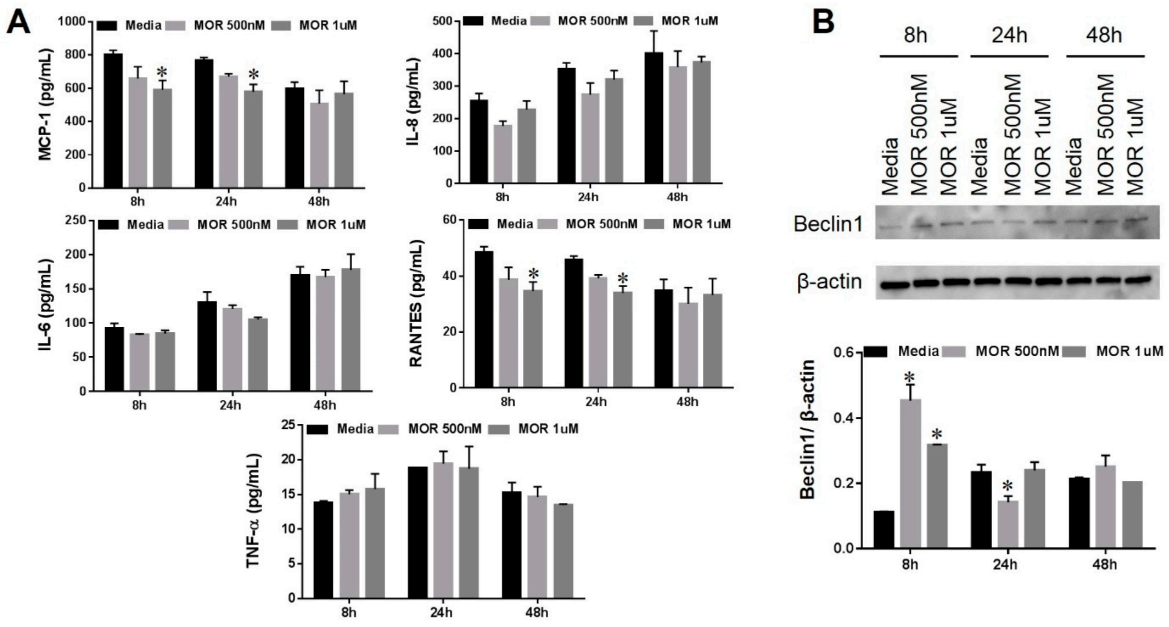

Figure 2. Effect of morphine on Beclin1 expression and inflammation in human astrocytes. **(A)** Cell culture supernatants were used to detect the indicated cytokines and chemokines by ELISA. Values were determined from standard curves and are presented as the mean ± the S.E.M. of three independent experiments ($p<0.05^*$ vs. Media). **(B)** Cell lysates from astrocytes with the indicated treatments were subjected to immunoblotting with antibodies to Beclin1. Densitometry was performed for quantification, and the ratios of each protein to β-actin are presented graphically. Error bars show the standard error of the mean (S.E.M.) of three independent experiments ($p<0.05^*$ vs. Media).

Table 1. Treatment with rapamycin and transfection with siBeclin1 modulate cell survival related gene

Gene	HIV-1	HIV-1+ MOR	HIV-1+ RAP	HIV-1+ RAP+MOR	HIV-1+ siBeclin1	HIV-1+ siBeclin1+MOR
BAK1	1.62	2.5669	2.7934	-1.0928	7.0911	13.0321
CASP8	-1.5735	1.1892	1.0367	-1.7029	2.0505	5.4039
GABARAPL2	-1.105	-1.181	1.7802	1.1266	2.1376	3.6402
RAB24	2.0792	4.1411	3.2761	1.3491	1.46	13.5855
TNFSF10	-1.105	-1.7053	1.2941	-1.0126	11.9257	9.9452
EIF4G1	-1.351	1.454	-3.0272	-1.4025	-2.7397	-2.3718

Autophagy-related gene expression were measured using qRT-PCR array assays. Of the 48 genes related to the autophagy pathway, only genes that showed a significant fold-change respective to control cells were selected. Several pro-apoptotic genes, including BAK1 and CASP8 were detected in lysates from cells transfected with siBeclin1. The upregulation was further increased when silencing of Beclin1 was combined with morphine. The indicated cell survival related gene expression levels were measured by gene array across the indicated treatment groups. Data are presented as fold of change relative to control cells.
